# A Spanish Adaptation of the Computer and Mobile Device Proficiency Questionnaires (CPQ and MDPQ) for Older Adults

**DOI:** 10.3389/fpsyg.2019.01165

**Published:** 2019-05-31

**Authors:** Carmen Moret-Tatay, María José Beneyto-Arrojo, Eugenia Gutierrez, Walter R. Boot, Neil Charness

**Affiliations:** ^1^Departamento de Metodología, Psicología Básica y Psicología Social, Universidad Católica de Valencia San Vicente Mártir, Valencia, Spain; ^2^Departamento de Personalidad, Evaluación e Intervención Terapéutica, Universidad Católica de Valencia San Vicente Mártir, Valencia, Spain; ^3^Department of Psychology, Florida State University, Tallahassee, FL, United States

**Keywords:** mobile device proficiency, computer proficiency, aging, digitalization, age related differences, digital divide, Spanish translation

## Abstract

Technology can help support the goal of many older adults to live independently, though cognitive, attitudinal, and other barriers often result in a “digital divide” in which older adults use and adopt new technology at a lower rate compared to younger adults. Due to the many potential benefits of technology it is not surprising that interest in tools that assess technology proficiency among older adults has increased. These tools can help support older adult technology research and training. However, to understand these issues more broadly, especially cross-cultural determinants of technology proficiency, translated, validated, and standardized measures of proficiency are necessary. For example, according to the last Eurobarometer (European Commission, 2015), Spain has experienced the largest increase in technology adoption among European Union nations in the past few years, indicating potential cultural mediation of technology adoption and use. To benefit the investigation of cross-cultural differences and their causes, we adapted the Mobile Device Proficiency Questionnaire (MDPQ) and Computer Proficiency Questionnaire (CPQ) for older adults in Spain, including the full and brief forms of each measure. Consistent with English versions of the questionnaires, the scales and their subscales were found to be reliable and valid measures of mobile device and computer proficiency in Spanish older adults. However, in contrast to earlier studies, the factor structure for both questionnaires simplified into two factors for the population under study. We conclude that the Spanish versions of the MDPQ and CPQ can be employed as useful tools for measuring mobile device and computer proficiency in the Spanish older adult population for research and training purposes.

## Introduction

In recent decades we have witnessed a rapid proliferation of information and communication technology (ICT). The use of technology that was once considered science fiction and economically infeasible is now practically a requirement to fully participate in modern society. However, not all groups have adopted some technologies to the same extent or have the same proficiency with respect to their use. In particular, seniors are commonly described as late adopters of technology in comparison to younger adults (Nikou, 2015; Hunsaker and Hargittai, 2018; Mitzner et al., 2018). Older adults have also been described as “digital immigrants” since in a relatively short period of time they had to immigrate from an analog world to a digital one (Yuan et al., 2016; Wang et al., 2019). Not surprisingly, this issue has been addressed from both a theoretical perspective and an applied one, because technology plays an increasingly important role in work, education, communication, and entertainment (e.g., Czaja et al., 2006; Charness and Boot, 2009). It is important to understand barriers to technology use and adoption, and potential facilitators, to ensure that individuals of all ages can reap the benefits of existing and emerging technologies, as described in recent meta-analysis (Scherer et al., 2019).

A variety of factors likely contribute to the observed age-related digital divide. Aging is associated with normative changes in general cognitive abilities (Salthouse, 2010), and cognitive abilities have been linked to the use of ICT (Czaja et al., 2006). Older adults' cognitive abilities shape their speed and accuracy interacting with technologies such as telephone menu systems, health applications, electronic medical records, and automatic teller machines (e.g., Czaja et al., 2010; Vaportzis et al., 2017). In other words, differences in access, use and/or impact of ICT may be driven in part, by declines in cognition with age (Klimova, 2016). In addition to cognition, there are attitudinal obstacles that prevent some older adults from adopting technology (Melenhorst et al., 2006) and problems in the design of these devices for older adults (Stronge et al., 2006). Technology anxiety and self-efficacy play important roles in shaping use and adoption (Czaja et al., 2006). These factors together help contribute to the observed age-related digital divide.

Moreover, as ICT spreads globally, differences among cultures have been observed in several countries (Straub et al., 1997). Some studies have found a strong positive relationship between technology adoption, education, infrastructure, and income that might have repercussions for economic growth (Quibria et al., 2003). More precisely, the use of a smartphone or computer varies considerably by country in Europe. According to the European Eurobarometer (European Commission, 2015), the largest increases have been seen in Spain, Italy, Croatia, and Hungary respectively, while other countries such as UK, Sweden, and Luxembourg that already exhibit a high adoption might be approaching a plateau. Moreover, one should bear in mind that cultural issues have also been related to the concept of digital divide. For this reason, we believe that more sophisticated measures that allow us to examine mobile and computer usage across countries are necessary.

The current study aims to adapt two new measures of the proficiency and use of mobile devices such as smartphones and tablets (Roque et al., 2016) and computers (Boot et al., 2015) for a Spanish population. This might shed light both in theoretical and applied fields, including the adaptation of training procedures based on an individual's current levels of proficiency. Therefore, questions of validity and reliability will be addressed. Internal consistency will be addressed across scales and versions, as well as age-related differences and convergent and divergent validity. The format of administration will be also taken into account. Finally, the factor structure will be re-examined in terms of exploratory factor analysis (EFA). In sum, we expect the new adaptation to be reliable (with a Cronbach's α greater than.80, as the original versions), valid (sensitive to group differences, predictive of variables related to frequency and length of technology use), and similarly structured in terms of factors as the original versions.

## Methods

### Participants

All participants were recruited from the Valencia community (in Spain) through an incidental sampling procedure. The sample selected consisted of 407 participants that ranged in age from 19 to 93 years (*M* = 53.07, *SD* = 23.19), and consisted of 39.5% men and 60.5% women (Table 1). The scoring scheme employed for the CPQ and MDPQ was the same as previously reported (Boot et al., 2015; Roque et al., 2016).

**Table 1:** Participant demographics in terms of age and gender.

		**Age**				**Gender**	
**Group**	***n***	**Min**.	**Max**.	**Mean**	***SD***	**Women (%)**	**Men (%)**
Young	132	19	34	26.03	4.81	72	28
Middle-aged	116	35	64	49.16	9.68	63.8	36.2
Older	159	65	93	78.17	5.90	49.1	50.9

### Procedure

Participants completed the questionnaires in a variety of settings either separately or in groups of three or four. In most cases the questionnaires were self-administered under the supervision of trained psychologists. Participants were asked for their preferred method to complete the questionnaire: on their own, and therefore online, or directly supervised and dictated by the psychologist. More precisely, all younger participants completed questionnaires online, while a total of 14.7% of the middle-aged and 62.3% of the older participants completed a dictated version of the questionnaires on paper. Even if the administration of this procedures might influence the response in the older adults, this was the most comfortable scenario for this group. Therefore, this was taken into account for the data analysis.

### Measures

For the adaptation of both the CPQ and MDPQ a back-translation process was performed as previously recommended in the literature (Muñiz et al., 2012). All original items were initially translated from English to Spanish by a native Spanish speaker with a fluent command of English, and then back-translated from Spanish to English by another bilingual professional. The result was discussed with native English speakers to identify potentially mistranslated items. For the few items that were identified, an alternative English to Spanish translation was made to preserve the meaning of the item. Both questionnaires employed a number of 5-point scales evaluating six domains for computer use and eight activity domains for mobile device use. Responses (1 = never tried, 2 = not at all, 3 = not very easily, 4 = somewhat easily, 5 = very easily) were averaged across subscales and the proficiency measure was the result of summing all subscales. Furthermore, short forms of the cited questionnaires were also evaluated. The different subscales, consisting of nine items, were comprised of questions related to computer and mobile device basics, participants' proficiency performing Communication tasks, Data and File Storage tasks, Transfer of Information, use of the Internet and Calendar, Entertainment, and Privacy among others (see Supplementary Material).

Finally, as in Roque et al. (2016), a short technology survey was included asking how long participants had been using Computers/Mobile devices, as well as how many hours per week participants used Computers/Mobile devices. These measures were used to help address the validity of the two measures under the assumption that use is correlated with proficiency.

## Results

**Figure 1:**
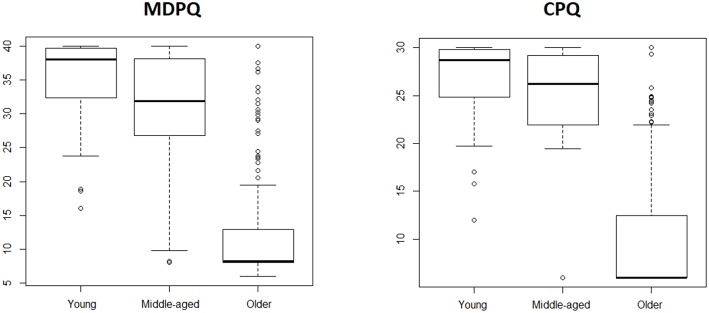
Box-and-whisker plot for the full versions of CPQ and MDPQ across age groups.

**Figure 2:**
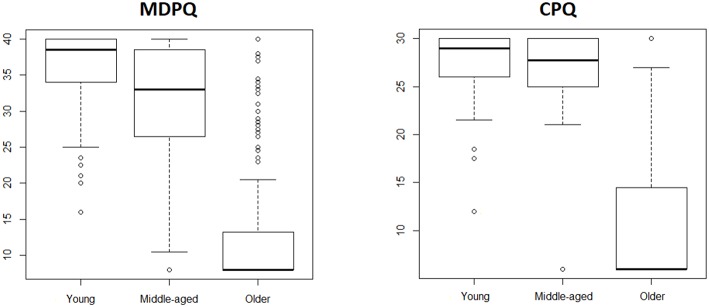
Box-and-whisker plot for the short versions of CPQ and MDPQ across age groups.

To address basic validity issues we first evaluated age differences. Based on the known digital divide between younger and older adults we predicted that older adults would score significantly lower compared to younger adults. This was confirmed, as depicted in Table 2 for the full versions, and in Table 3 for the shorter versions of the CPQ and MDPQ. In Figures 1, 2, box-and-whisker plot shows how the middle-aged adults presented higher variability.

**Table 2:** Descriptive statistics for both full versions of the MDPQ and CPQ.

		**Young**		**Middle-aged**		**Older**	
		**Mean**	***SD***	**Mean**	***SD***	**Mean**	***SD***
Mobile device	Mobile device basics	4.75	0.41	4.29	0.89	2.10	1.30
	Communication	4.48	0.76	3.73	1.32	1.62	1.20
	Data and file storage	4.36	0.92	3.42	1.50	1.44	1.02
	Internet	4.60	0.70	3.91	1.31	1.65	1.21
	Calendar	4.32	1.20	3.54	1.54	1.40	1.04
	Entertainment	4.52	0.75	3.63	1.36	1.59	1.11
	Privacy	4.37	0.81	3.50	1.33	1.57	1.09
	Troubleshooting and software management	4.60	0.70	3.78	1.34	1.63	1.17
Computer	Computer basics	4.69	0.46	4.12	1.35	1.90	1.42
	Printing	4.49	0.74	4.08	1.39	1.78	1.36
	Communication	4.75	0.49	4.03	1.36	1.71	1.25
	Internet	4.83	0.49	4.19	1.38	1.75	1.24
	Calendar	3.71	1.74	3.26	1.71	1.29	0.80
	Entertainment	4.70	0.64	3.79	1.47	1.58	1.13
Mobile device proficiency		36.02	5.01	29.80	9.63	13.13	8.86
Computer proficiency		27.17	3.40	23.47	7.85	10.69	7.49

**Table 3:** Descriptive statistics for both short versions of the MDPQ and CPQ.

		**Young**		**Middle-aged**		**Older**	
		**Mean**	**SD**	**Mean**	**SD**	**Mean**	**SD**
Mobile device	Mobile device basics	4.85	0.42	4.54	0.83	2.20	1.56
	Communication	4.68	0.73	4.09	1.43	1.71	1.39
	Data and file storage	4.39	0.99	3.49	1.55	1.47	1.08
	Internet	4.64	0.69	4.08	1.39	1.78	1.44
	Calendar	4.29	1.28	3.56	1.57	1.40	1.04
	Entertainment	4.60	0.86	3.62	1.49	1.48	1.06
	Privacy	4.38	0.96	3.42	1.45	1.56	1.12
	Troubleshooting and software management	4.64	0.82	3.77	1.44	1.52	1.15
Computer	Computer basics	4.95	0.29	4.39	1.42	2.08	1.66
	Printing	4.19	1.02	4.13	1.41	1.78	1.37
	Communication	4.94	0.34	4.39	1.42	1.92	1.57
	Internet	4.85	0.48	4.32	1.41	1.98	1.60
	Calendar	3.71	1.75	3.32	1.75	1.27	0.83
	Entertainment	4.62	0.80	4.05	1.52	1.66	1.28
Mobile device proficiency		36.47	4.85	30.56	9.58	13.13	8.86
Computer proficiency		27.27	3.22	23.47	7.85	10.69	7.49

### Reliability of the CPQ and MDPQ

Cronbach's α was used to assess the reliability of the both MDPQ and CPQ. Overall, the scale demonstrated excellent reliability (Cronbach's α = 0.99 for both questionnaires), with subscale reliabilities ranging from 0.71 to 0.99 for MDPQ, and from 0.51 to 0.98 for CPQ across age groups, see Table 4.

**Table 4:** Internal consistency for the MDPQ and CPQ.

		**Young**	**Middle-aged**	**Older**
Mobile device	Mobile device basics	0.801	0.921	0.964
	Communication	0.850	0.949	0.984
	Data and file storage	0.754	0.924	0.975
	Internet	0.870	0.946	0.976
	Calendar	0.908	0.956	0.955
	Entertainment	0.715	0.897	0.947
	Privacy	0.670	0.866	0.957
	Troubleshooting and software management	0.834	0.935	0.971
	Total	0.956	0.986	0.993
Computer	Computer basics	0.514^*^	0.945	0.975
	Printing	0.815	0.972	0.984
	Communication	0.779	0.960	0.974
	Internet	0.852	0.976	0.953
	Calendar	0.978	0.967	0.903
	Entertainment	0.633	0.883	0.884
	Total	0.914	0.988	0.989

* *After conducting the Cronbach's Alpha if Item Deleted, we realize that if some items in the Basics of a Computer would be taken out (e.g., “Use a trackball”) alphas would be better (In that case alpha = 0.67)*.

### Validity of the CPQ and MDPQ

In order to examine convergent and divergent validity, Pearson correlation coefficients were computed across questionnaires, and variables of length and frequency of use. Age was also considered in the analysis, being reliably negatively correlated with MDPQ, MDPQ-16, CPQ, and CPQ-12. Table 5 depicts the relationship among variables. As expected, the corresponding full and shorter version of the same questionnaire were more highly correlated than different questionnaires. This result was also similar for the relationship with length and frequency of use, which showed higher validity for the pertinent questionnaire. In contrast to the case for the original American sample, there is less differentiation in prediction (convergent/divergent validity) between the CPQ and the MDPQ and their respective use behaviors. This finding may indicate some combination of cultural differences and factor structure differences.

**Table 5:** Pearson's coefficients among MDPQ, CPQ, MDPQ-16, CPQ-12, Frequency and length of use.

	**Young**				**Middle-aged**				**Older**				**Total**			
	**MDPQ**	**MDPQ-16**	**CPQ**	**CPQ-12**	**MDPQ**	**MDPQ-16**	**CPQ**	**CPQ-12**	**MDPQ**	**MDPQ-16**	**CPQ**	**CPQ-12**	**MDPQ**	**MDPQ-16**	**CPQ**	**CPQ-12**
Age	−0.21^*^	−0.19^*^	−0.15	−0.10	−0.39^**^	−0.40^**^	−0.35^**^	−0.39^**^	−0.62^**^	−0.62^**^	−0.67^**^	−0.67^**^	−0.82^**^	−0.82^**^	−0.80^**^	−0.78^**^
Mobile length of use	0.15	0.13	0.06	0.03	0.30^**^	0.31^**^	0.19^*^	0.20^*^	0.58^**^	0.57^**^	0.52^**^	0.52^**^	0.72^**^	0.72^**^	0.68^**^	0.69^**^
Frequency mobile use	0.26^**^	0.24^**^	0.08	0.01	0.39^**^	0.41^**^	0.34^**^	0.35^**^	0.83^**^	0.82^**^	0.79^**^	0.79^**^	0.81^**^	0.82^**^	0.78^**^	0.78^**^
Computer length of use	−0.09	−0.09	−0.02	−0.00	0.84^**^	0.84^**^	0.86^**^	0.88^**^	0.80^**^	0.79^**^	0.93^**^	0.93^**^	0.87^**^	0.87^**^	0.91^**^	0.92^**^
Computer frequency	0.11	0.09	0.17^*^	0.11	0.78^**^	0.79^**^	0.84^**^	0.85^**^	0.80^**^	0.79^**^	0.92^**^	0.91^**^	0.87^**^	0.87^**^	0.91^**^	0.91^**^
MDPQ	1	0.97^**^	0.76^**^	0.73^**^	1	0.99^**^	0.92^**^	0.91^**^	1	0.99^**^	0.91^**^	0.89^**^	1	0.99^**^	0.96^**^	0.94^**^
MDPQ-16	0.97^**^	1	0.73^**^	0.71^**^	0.99^**^	1	0.92^**^	0.90^**^	0.99^**^	1	0.91^**^	0.89^**^	0.99^**^	1	0.95^**^	0.95^**^
CPQ	0.76^**^	0.73^**^	1	0.96^**^	0.92^**^	0.92^**^	1	0.98^**^	0.91^**^	0.91^**^	1	0.99^**^	0.96^**^	0.95^**^	1	0.99^**^
CPQ-12	0.73^**^	0.71^**^	0.96^**^	1	0.91^**^	0.90^**^	0.98^**^	1	0.89^**^	0.89^**^	0.99^**^	1	0.94^**^	0.95^**^	0.99^**^	1

* p < 0.05;

** *p < 0.001*.

**Figure 3:**
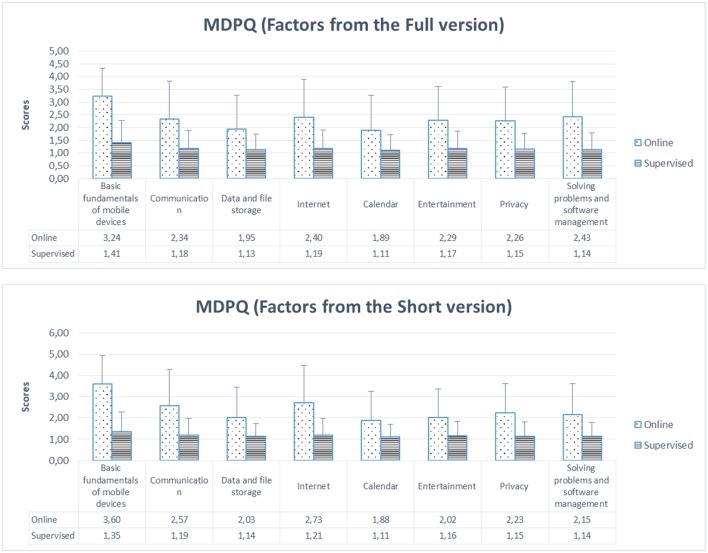
Mean for each MDPQ factor (both full and short versions) and their SD (standard deviation) across format of administration: online vs. supervised (dictated).

**Figure 4:**
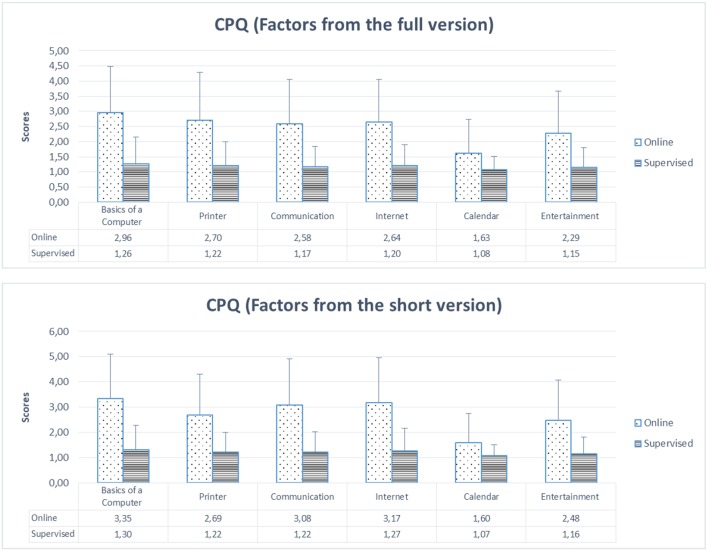
Mean for each CPQ factor (both full and short versions) and their SD (standard deviations) across format of administration: online vs. supervised (dictated).

Moreover, the validity in the older group was tested across format of administration: online vs. supervised, or better to say, dictated. As depicted in Figures 3, 4, the online group scored higher than the other group, and these differences were significant for all conditions as indicated by independent samples *t*-tests (all *p* < 0.0001).

### Factor Structure

As young participants seem to show a ceiling effect as in the previous literature (Roque et al., 2016), and older adults presented low variance, MDPQ and CPQ structures were examined under a factor analysis conducted on older and middle adult data together using Varimax rotation. For the MDPQ, two factors accounted for 88.21% of the variation in the data. A similar structure was found for the CPQ with an explained variance that reached an 84.06%. For the MDPQ, the Bartlett's test of sphericity was *p* < 0.001, with a value of chi-square 23958.48 (*df* = 1035), and the sample index value of Kaiser-Meyer-Olkin (KMO) was 0.961. For the CPQ, the Bartlett's test of sphericity was *p* < 0.001 with a value of chi-square 20488.76 (*df* = 528) and the sample index value of KMO was 0.964. Finally, the whole database was analyzed together (all groups). Tables 6, 7 depict the factor loading for both the MDPQ and CPQ. Only two factor solution were uncovered for each. For the MDPQ it seems one factor was related to mobile device fundamentals (e.g., turning device on and off, using the touchscreen keyboard, adjusting volume) and the other factor was more related to advanced functions and features (sending emails, using instant messenger, updating software). The CPQ was relatively unidimensional, with one large factor representing most computer functions and features, and the second small dimension relating largely to electronic calendar use. These analyses are unlike those initially reported by Roque et al. (2016) and Boot et al. (2015), which found a factor structure that more closely matched the intended subscales of each measure.

**Table 6:** Factor loading for the CPQ.

	**Factor**	
	**1**	**2**
To turn on and off a computer	**0.893**	0.352
Use the keyboard to write/type	**0.899**	0.359
Use a trackball	0.436	**0.647**
Use a mouse	**0.900**	0.357
Adjust the volume of the speakers of the computer	**0.881**	0.407
Adjust the size of the text on the screen	**0.781**	0.483
Print documents	**0.895**	0.372
Print photographs	**0.811**	0.417
Put paper in the printer	**0.895**	0.350
Change the ink for the printer	**0.805**	0.407
Fix the printer when it produces a bunch of papers	**0.777**	0.464
Open emails	**0.902**	0.386
Send emails	**0.899**	0.395
Send the same email to multiple people at the same time	**0.845**	0.450
Storing emails in an address book or a contacts list	**0.682**	0.579
See photographs that were sent through an email	**0.892**	0.401
Send photographs through email	**0.885**	0.420
Chat using a chat room on the internet	0.574	**0.604**
Chat using instant messaging	**0.665**	0.569
Publish messages online through a blog, Facebook, twitter, or other online forms	**0.718**	0.545
Use websites to look stuff up (example, Google)	**0.897**	0.381
Find information about resources in the community on the internet	**0.869**	0.389
Find information about my hobbies or interests on the internet	**0.894**	0.384
Read the news online	**0.886**	0.369
Buy things on the internet	**0.720**	0.536
Bookmark pages on the internet to return to and find again at a later date (for example, to favorite a page)	**0.761**	0.524
save documents and images so that you find on the internet	**0.827**	0.471
Use the computer to input events and dates in a calendar	0.307	**0.905**
Check the date and time of previous and upcoming appointments	0.303	**0.910**
Send myself alerts to remind me about events and dates	0.292	**0.894**
Use the computer to play games	0.565	**0.639**
Use the computer to watch movies and videos	**0.828**	0.461
Use the internet to listen to music	**0.806**	0.468

*The bold value significant item loading value representing the weigh*.

**Table 7:** Factor loading for the MDPQ.

	**Factor**	
	**1**	**2**
Turn on and off a device	0.288	**0.878**
Charge the device when the battery is low	0.208	**0.880**
Scroll through the menus on the screen using the touchscreen	0.451	**0.818**
Use the keyboard on the screen to write	0.435	**0.840**
Copy and paste text using the touchscreen	**0.717**	0.564
Adjust the volume of the device	0.449	**0.830**
Adjust the brightness of the device	0.649	**0.670**
Adjust the size of the text	**0.696**	0.492
Connect up to wifi	0.679	**0.680**
Open emails	0.649	**0.712**
Send emails	**0.669**	0.675
Send the same email to different people at the same time	**0.726**	0.570
Store email addresses and send emails to people from my contact list	**0.708**	0.394
Look at photos sent to me by email	**0.655**	0.643
Send photos by email	**0.691**	0.614
Publish messages on online social networks such as (Facebook Twitter Instagram Google Plus)	**0.744**	0.522
Use instant messaging (for example AIM Yahoo Messenger MSN Messenger)	**0.753**	0.425
Use video messaging such as Skype Google Hangout FaceTime	**0.810**	0.335
Transfer information such as music photos documents from my mobile device to my computer	**0.777**	0.493
Transfer information from my computer to my mobile device	**0.813**	0.414
Save information with services that allow me to see my information on whatever device I want such as Dropbox Google drive Microsoft Onedrive	0.812	0.384
Use search engines such as google and Bing	0.633	**0.723**
Find information about local resources in the community online	0.622	**0.666**
Find information about my hobbies and interests online	0.628	**0.727**
Find medical information online	**0.644**	0.635
Read the news online	0.656	**0.662**
Shop online	**0.784**	0.425
Bookmark my favorite web pages so that I can return to them at a later time	**0.795**	0.443
Save images and texts that I find online	**0.782**	0.477
Create events and make the date in the calendar	**0.800**	0.421
Check the date and time of upcoming and past appointments	**0.770**	0.424
Make alerts to remind me of events and dates	**0.792**	0.436
Use the phone to go on online stores to find games and other forms of entertainment store or Google play store	**0.790**	0.421
Watch movies and videos	**0.679**	0.582
Listen to music	**0.697**	0.594
Read a book	**0.743**	0.332
Take photos and videos	0.611	**0.710**
Create a password to block/unblock a mobile device	**0.739**	0.540
Delete photos and videos stored on the device	0.652	**0.707**
Delete the search history and temporary files	**0.777**	0.376
Reset to factory settings deleting all the account's information	**0.797**	0.281
Restart the device when its blocked or not functioning well	0.560	**0.708**
Update games and other applications	**0.757**	0.536
Close games and other applications	**0.740**	0.599
Delete games and other applications	**0.742**	0.574
Update the device's software	**0.770**	0.444

*The bold value significant item loading value representing the weigh*.

## Discussion and Conclusions

We have recently witnessed a revolution in one particular technological development. Mobile and Computer communication is an emerging technology that has been described as shaping the way that we process information and think (Dufau et al., 2011; Wilmer et al., 2017). Furthermore, age-related differences have been described in this field, relating use of ICT with cognition and maintaining independence (Charness and Boot, 2009). For this reason, measurement tools that might shed light on the adoption of ICT are of interest. In particular, in a population like the Spanish one, where, to our knowledge, if the measures is rather scarce, such tools would be very useful. For this reason, a Spanish adaptation of two of the most widespread questionnaires in this field was proposed.

First, a back-translation was carried out for the MDPQ and CPQ questionnaires. After this step, the questionnaires of interest were administered to different age groups. As expected, older participants exhibited lower scores than the younger ones. However, the scores were even lower than reported in the previous literature carried out in United States. This could indicate cultural differences among populations. Here it is important to bear in mind that Spain, as indicated in the European report has presented a remarkable increase in technology adoption. Nevertheless, a similar tendency on scores was found to Roque et al. (2016), where subscales related to more complex actions such as calendar, managing privacy settings, and files demonstrated greater variability and lower scores.

Both questionnaires presented excellent internal consistency and validity; however, differences in terms of structure were noteworthy. As mentioned before, this might be due to the moment in which the Spanish use of ICT is lower, in comparison with other countries such as the USA. In this way, it seems not surprising that the most popular way to participate in the study, by the older adults, was under dictated supervision, not online. More precisely, a large body of research has hypothesized that the adoption of new technologies would be stronger in more globalized cultures (Zahir et al., 2002). On the other hand, some gender differences occurred, specifically for the older group. Usually, women outnumber men in old age, but, in this sample, older men were overrepresented. Moreover, in a qualitative way, it is possible to state that older men showed more willingness to participle in the study. It also could account for some of the differences in factor structure if technology adoption in older groups follows the path seen early in the USA. Here, men adopted computer technology earlier than women but the digital divide disappeared over time. If older adults in Spain are just now adopting technology there may be a similar divide in competence.

Finally, there are several shortcomings in the present study. First, the participants were selected through non-probability sampling, which can introduce distortions in the results, for instance in male vs. female representation. Secondly, self-report bias may occur through the use of different sources employed in the data collection, online or through paper. This action was employed because of the rejection from most of the older participants on completing the questionnaire online. For this reason, questionnaires on paper for both face-to-face and telephone recruitment were employed. Future lines of research should compare both formats across age groups.

In sum, we present an adaptation of the MDPQ and CPQ for the Spanish population that might be of interest not only for examining the adoption of technologies in the Spanish elderly, but also for cross-cultural studies that allow us to compare different populations. From a theoretical level, the factor structure differences observed across studies contributed to the advancement of research models on aging and associated variables inherent to technology adoption. On a practical level, these translations offer a new tool for empirical studies. In particular, individual difference in proficiency might lead to different technology use patterns. Therefore, these results might reflect reasons of underuse or even inappropriate application of devices for training proposes, as indicated by Roque et al. (2016). In sum, these tools might shed light on participant's skill level before starting training. Moreover, questionnaire scores might be used to organize groups of older adults with similar proficiency, thereby optimizing technology training activity that can promote independence in older adults. Future lines of research could also explore the relationship between technology proficiency and cognitive status including the role of bilingualism and engagement in intellectual activities (Neville et al., 2018).

## Ethics Statement

All participants completed a written informed consent and the research was approved by the ethical committee at the Universidad Católica de Valencia, San Vicente Mártir.

## Author Contributions

WB and NC conceived of the presented idea. CM-T, WB and NC conceived the back translation and planned the data recruitment. CM-T, MB-A and EG recruited the data. CM-T, WB and NC drafted the manuscript.

### Conflict of Interest Statement

The authors declare that the research was conducted in the absence of any commercial or financial relationships that could be construed as a potential conflict of interest.
