# Supplementary material for: A Spanish Adaptation of the Computer and Mobile Device Proficiency Questionnaires (CPQ and MDPQ) for Older Adults
Source: Front Psychol. 2019 May 31;10:1165. doi: 10.3389/fpsyg.2019.01165 (PMC6554441; doi:10.3389/fpsyg.2019.01165)
Supplement: Supplementary file 1 [file Data_Sheet_1.PDF]

CUESTIONARIO SOBRE COMPETENCIAS CON EL ORDENADOR

Nota : Las preguntas que se han conservado de la versión breve del CCO (CPQ -12) están marcadas con un asterisco

Este cuestionario pregunta acerca de su capacidad para llevar a cabo una serie de tareas con el ordenador. Por favor, conteste cada pregunta, marcando una X en la casilla que sea más apropiada. Si usted no ha intentado realizar una tarea o no sabe lo que es, por favor marque "NUNCA LO INTENTÉ", independientemente de si usted piensa que puede ser capaz o no de realizar la tarea.

1 Fundamentos básicos del ordenador

Puedo	Nunca lo intenté	En absoluto	No fácilmente	Fácilmente en cierto modo	Muy fácilmente
a) Encender y apagar un ordenador					
b) Utilizar un teclado de ordenador para escribir*					
c) Usar una bola de seguimiento (*trackball*)					
d) Usar un ratón*					
e) Ajustar el volumen de los altavoces del ordenador					
f) Ajustar el tamaño del texto en la pantalla					

2. Impresora

Puedo	Nunca lo intenté	En absoluto	No fácilmente	Fácilmente en cierto modo	Muy fácilmente
a) Imprimir documentos					
b) Imprimir fotografías					
c) Cargar papel en la impresora					
d) Cargar tinta en la impresora*					

e) Arreglar la impresora cuando se produzcan atascos de papel*					

3. Comunicación

Puedo	Nunca lo intenté	En absoluto	No fácilmente	Fácilmente en cierto modo	Muy fácilmente
a) Abrir correos electrónicos (*emails*)*					
b) Enviar correos electrónicos (*emails*)*					
c) Enviar el mismo correo electrónico (*email*) a varias personas en el mismo momento					
d) Almacenar correos electrónicos (*emails*) en una libreta de direcciones o lista de contactos					
e) Ver fotografías enviadas por correo electrónico (*email*)					
f) Enviar fotografías por correo electrónico (*email*)					
g) Chatear usando una sala de chat en internet					
h) Chatear usando mensajes instantáneos					
i) Publicar mensajes en internet (por ejemplo en Blogs, Facebook, Twitter, Fórums online)					

4. Internet

Puedo	Nunca lo intenté	En absoluto	No fácilmente	Fácilmente en cierto modo	Muy fácilmente

a) Usar motores de búsqueda (ejemplo, Google)					
b) Encontrar información sobre recursos locales de la comunidad en Internet*					
c) Encontrar información acerca de mis aficiones e intereses en Internet*					
d) Leer las noticias en internet					
e) Hacer compras en internet					
f) Marcar páginas web para volver a encontrarlas más tarde (por ejemplo, agregar a favoritos)					
g) Guardar texto e imágenes que encuentro en internet					

5. Calendario

Puedo	Nunca lo intenté	En absoluto	No fácilmente	Fácilmente en cierto modo	Muy fácilmente
a) Utilizar un ordenador para introducir eventos y citas en un calendario*					
b) Comprobar la fecha y hora de próximas y anteriores citas*					
c) Instalarme alertas para recordarme eventos y citas					

6. Entretenimiento

Puedo	Nunca lo intenté	En absoluto	No fácilmente	Fácilmente en cierto modo	Muy fácilmente
a) Usar el ordenador para jugar a juegos					
b) Utilizar un ordenador para ver películas y videos*					
c) Utilizar un ordenador para escuchar música*					
