# Supplementary material for: A Spanish Adaptation of the Computer and Mobile Device Proficiency Questionnaires (CPQ and MDPQ) for Older Adults
Source: Front Psychol. 2019 May 31;10:1165. doi: 10.3389/fpsyg.2019.01165 (PMC6554441; doi:10.3389/fpsyg.2019.01165)
Supplement: Supplementary file 2 [file Data_Sheet_2.PDF]

Cuestionario de Aptitud de dispositivos móviles

Este cuestionario pregunta acerca de su capacidad para llevar a cabo una serie de tareas con un dispositivo móvil.

¿Qué es un dispositivo móvil?
Un dispositivo móvil es un dispositivo que le permite realizar muchas de las mismas tareas que un ordenador estándar, pero sin el uso de un teclado físico y el ratón. En su lugar, estos dispositivos utilizan una pantalla táctil así como su interfaz entre el usuario y los programas equipo (llamados App- abreviación para Aplicaciones).

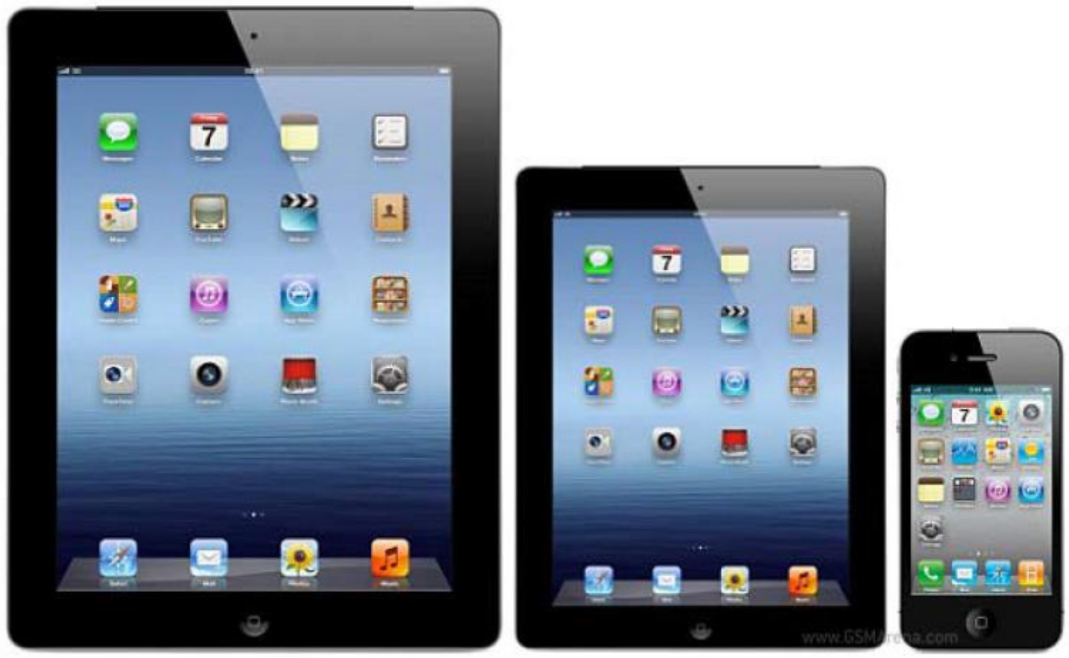

Los dispositivos móviles vienen en muchos tamaños. Como se muestra arriba, hay dos tamaños diferentes de tableta (*Tablet*), así como un teléfono inteligente (*Smartphone*). Estos son los tipos de dispositivos que nos interesan.

Por favor, conteste cada pregunta marcando con una X en la casilla que sea más apropiada.

Si usted no ha intentado realizar una tarea o no sabe lo que es, por favor marque "NUNCA LO INTENTÉ", independientemente de si usted piensa que puede ser capaz o no de realizar la tarea. **Recuerde, usted está valorando su capacidad de realizar cada una de estas tareas específicamente utilizando un dispositivo móvil (tableta o teléfono inteligente, o lo que es lo mismo, *Tablet o smartphone*).**

1 Fundamentos básicos de los dispositivos móviles

Usando un dispositivo móvil<br><br>Puedo	Nunca lo intenté (1)	En absoluto (2)	No fácilmente (3)	Fácilmente en cierto modo (4)	Muy fácilmente (5)
a) Encender y apagar el dispositivo					
b) Cargar el dispositivo cuando la batería esta baja					
c) Navegar en los menús de pantalla usando la pantalla táctil					
d) Usar el teclado en pantalla para escribir					
e) Copiar y pegar texto usando la pantalla táctil					
f) Ajustar el volumen del dispositivo					
g) Ajustar el brillo de la pantalla					
h) Ajustar el tamaño de texto					
i) Conectarse a una red Wi-Fi					

2. Comunicación

Usando un dispositivo móvil<br><br>Puedo	Nunca lo intenté	En absoluto	No fácilmente	Fácilmente en cierto modo	Muy fácilmente
a) Abrir emails					
b) Enviar emails					

c) Enviar el mismo email a varias personas en el mismo momento					
d) Almacenar correos electrónicos (*emails*) en una libreta de direcciones o lista de contactos					
e) Ver fotografías enviadas por correo electrónico (*email*)					
f) Enviar fotografías enviadas por correo electrónico (*email*)					
g) Publicar mensajes en redes sociales (por ejemplo en Facebook, Twitter, Instagram, Google Plus)					
h) Usar mensajes instantáneos (por ejemplo, AIM, Yahoo, Messenger, MSN Messenger)					
i) Usar mensajes de video (por ejemplo, Skype, Google, Hangout, FaceTime)					

3. Datos y almacenamiento de archivos

Usando un dispositivo móvil<br><br>Puedo	Nunca lo intenté	En absoluto	No fácilmente	Fácilmente en cierto modo	Muy fácilmente
a) Trasladar información (archivos como música, fotografías, documentos) en mi dispositivo móvil a mi ordenador					
b) Trasladar					

información (archivos como música, fotografías, documentos) en mi ordenador a mi dispositivo móvil					
c) Almacenar información con un servicio que me permita ver mis archivos desde cualquier lugar (por ejemplo, Dropbox, Google, Drive, Microsoft onedrive)					

4. Internet

Usando un dispositivo móvil<br><br>Puedo	Nunca lo intenté	En absoluto	No fácilmente	Fácilmente en cierto modo	Muy fácilmente
a) Usar motores de búsqueda (ejemplo, Google, Bing)					
b) Encontrar información sobre recursos locales de la comunidad en Internet					
c) Encontrar información acerca de mis aficiones e intereses en Internet					
d) Encontrar información médica en internet					
e) Leer las noticias en internet					
f) Hacer compras en internet					
g) Marcar páginas web para volver a encontrarlas más tarde (por ejemplo, agregar a favoritos)					
h) Guardar texto e imágenes					

que encuentro en internet					

5. Calendario

Usando un dispositivo móvil<br><br>Puedo	Nunca lo intenté	En absoluto	No fácilmente	Fácilmente en cierto modo	Muy fácilmente
a) Crear eventos y citas en un calendario					
b) Comprobar la fecha y hora de próximas y anteriores citas					
c) Instalar alertas para recordarme eventos y citas					

6. Entretenimiento

Usando un dispositivo móvil/Tableta (Tablet)<br><br>Puedo	Nunca lo intenté	En absoluto	No fácilmente	Fácilmente en cierto modo	Muy fácilmente
a) Utilizar el dispositivo de "tienda en línea" (*online store*) para encontrar juegos y otras formas de entretenimiento (por ejemplo, usar Apple app Store o Google Play Store)					
b) Ver películas y videos					
c) Escuchar música					
d) Leer un libro					
e) Hacer fotografías vídeos					

7 Privacidad

Usando un dispositivo móvil/Tableta (Tablet)<br><br>Puedo	Nunca lo intenté	En absoluto	No fácilmente	Fácilmente en cierto modo	Muy fácilmente
a) Instalar una contraseña para bloquear/desbloquear el dispositivo					
b) Eliminar fotografías y videos almacenados en el dispositivo					
c) Eliminar todo el historial de navegación en Internet y archivos temporales					
d) Restablecer los ajustes de fábrica del dispositivo, borrando toda la información de la cuenta					

8. Solución de problemas y gestión de software

Usando un dispositivo móvil/Tableta (Tablet)<br><br>Puedo	Nunca lo intenté	En absoluto	No fácilmente	Fácilmente en cierto modo	Muy fácilmente
a) Reiniciar el dispositivo cuando se bloquea o no funciona bien					
b) Actualizar juegos y otras aplicaciones					
c) Cerrar juegos y otras aplicaciones					
d) Eliminar juegos y otras aplicaciones					
e) Actualizar el software del dispositivo					
